# Social determinants of vulnerability in the population of reproductive age: a systematic review

**DOI:** 10.1186/s12889-022-13651-6

**Published:** 2022-06-24

**Authors:** Lindsey van der Meer, Lisa S. Barsties, Leonie A. Daalderop, Adja J. M. Waelput, Eric A. P. Steegers, Loes C. M. Bertens

**Affiliations:** 1 Department of Obstetrics and Gynaecology, Erasmus MC, University Medical Center Rotterdam, PO Box 2040, Rotterdam, 3000 CA The Netherlands; 2 DRIFT – Dutch Research Institute for Transitions, Erasmus University Rotterdam, Rotterdam, The Netherlands

**Keywords:** Social determinants, Epidemiology, Population health, Vulnerable populations, Preconception care

## Abstract

**Background:**

The health of an (unborn) child is largely determined by the health and social determinants of its parents. The extent to which social determinants of parents or prospective parents affect their own health depends partly on their coping or resilience abilities. Inadequate abilities allow negative effects of unfavourable social determinants to prevail, rendering them vulnerable to adverse health outcomes. Addressing these determinants in the reproductive-aged population is therefore a key approach in improving the health of the future generation. This systematic review aims to synthesise evidence on social determinants of vulnerability, i.e., inadequate coping or low resilience, in the general population of reproductive age.

**Methods:**

The databases EMBASE, Medline, PsycINFO, CINAHL, Google Scholar, Web of Science, and Cochrane Library, were systematically searched from database inception to December 2th 2021.

Observational studies examining social determinants and demographics in relation to vulnerability among the general population of reproductive age (men and women aged 18-40 years), conducted in a high-income country in Europe or North America, Australia or New Zealand were eligible for inclusion. Relevant data was extracted from each included article and findings were presented in a narrative and tabulated manner.

**Results:**

We identified 40,028 unique articles, of which 78 were full text reviewed. Twenty-five studies were included, of which 21 had a cross-sectional study design (84%). Coping was the most frequently assessed outcome measure (*n* = 17, 68%). Thirty social determinants were identified. Overall, a younger age, lower socioeconomic attainment, lack of connection with the social environment, and adverse life events were associated with inadequate coping or low resilience.

**Conclusions:**

This review shows that certain social determinants are associated with vulnerability in reproductive-aged individuals. Knowing which factors make people more or less vulnerable carries health-related implications. More high-quality research is needed to obtain substantial evidence on the strength of the effect of these social conditions in this stage of life.

**Supplementary Information:**

The online version contains supplementary material available at 10.1186/s12889-022-13651-6.

## Background

There exists a social gradient in health that is visible throughout the entire life course. Socially disadvantaged individuals are at higher risk of adverse health outcomes compared to those who are socially privileged [1, 2]. Individuals’ social conditions can affect their health in different ways. For example, individuals who face greater social disadvantage often have fewer resources, experience more stress, or live in disadvantaged neighbourhoods. The interplay between such social conditions (i.e., social determinants) increases the risk of poor health [2–4].

The World Health Organization (WHO) conceptualises social determinants as ‘the conditions in which people are born, grown, live, work and age’ [5]. Their Social Determinants Of Health (SDOH) framework elaborates on micro and macro level social determinants and their influence on the health of individuals, emphasizing the need to include these determinants in health research. Macro level determinants, such as policies on health, education, or the labour market, influence the socioeconomic stratification in societies. In turn, individuals’ socioeconomic position (micro level) influences their daily environment and exposures [6].

Social determinants not only affect the health of the current generation but also that of future generations [7, 8]. For example, parental health and social determinants can affect foetal development. Suboptimal foetal development has repercussions for health at birth, during child- and adulthood [8]. Poor health during childhood is a precursor for lower educational attainment and less socioeconomic and social opportunities during adulthood, affecting labour market participation and social engagement [9]. Given these potentially far-reaching implications, it is particularly important to place a greater emphasis on improving the preconception health of individuals of reproductive age by addressing their social determinants.

The extent to which social determinants impact the health of individuals of reproductive age partly depends on their coping and resilience abilities. Inadequate coping or low resilience allow negative effects of unfavourable determinants to prevail, increasing the risk of adverse health outcomes. An imbalance between exposure to adverse determinants and the ability to cope adequately or being resilient enough is often described as being vulnerable [10].

A comprehensive overview of what is currently known about the social determinants of vulnerability in the general population of reproductive age is lacking. Most research on vulnerability focuses on specific subpopulations, such as the elderly, children, or ethnic minorities [11, 12]. The general reproductive population is often considered to be a healthy subpopulation that experiences less adverse social determinants or can deal with them adequately [13, 14]. The aim of this review is to synthesise existing evidence on social determinants of vulnerability, defined as insufficient coping or resilience abilities, of the general population of reproductive age. Insights into these determinants can help to identify vulnerable individuals. Furthermore, interventions or policies can be better tailored to optimise the health and social conditions of this population group, which can have profound consequences for the health of the next generation.

## Methods

To conduct this systematic review, pre-specified methods were followed that are registered with PROSPERO (CRD42018090743). The PRISMA (Preferred Reporting Items for Systematic reviews and Meta-Analyses) Statement was used as a guideline for reporting [15].

### Eligibility criteria

Observational studies (i.e., cohort, case-control, and cross-sectional studies) assessing social or demographic determinants of coping or resilience were eligible for inclusion. Social determinants that were considered in this review related to the domains described by the Healthy People 2030 Framework of Social Determinants [16]. Of these domains we included: economic stability, education, neighbourhood and built environment, and social and community context (Table 1). Demographics (i.e., age, gender, and ethnicity) were included as determinants because they partly define the social position of individuals and therefore the social determinants they are exposed to [6].

**Table 1:** Social determinants, domains of interest

Domain	Examples
Economic stability	Socioeconomic status, (un)employment, poverty
Education	Educational level, literacy
Neighbourhood and built environment	Neighbourhood resources, housing quality, urbanisation degree
Social and community context	Stress, social support, social cohesion

Studies were eligible for inclusion when at least 50% of their study sample was of reproductive age, defined as being between the ages of 18 to 40 years, to capture all parents or prospective parents. The lower limit was set at 18 years old, to exclude minors whose social determinants may still be closely intertwined with their parents’ determinants. The upper limit was set at 40 years old, since advanced maternal or paternal age at conception is less common [17]. The criterion that at least 50% of the participants must be aged between 18 and 40 years for studies to be included was made after preliminary screening revealed that studies often refrain from providing detailed information about the age range or distribution. Therefore, studies that reported an age range or mean age with standard deviation that fell within our range of interest, 18-40 years, were eligible for inclusion.

Furthermore, studies that were conducted in high-income countries in Europe or North America, Australia, and New Zealand were considered eligible, ensuring that determinants that explicitly apply to low- and middle-income countries were not included. A country was defined as high-income, based on the World Bank Country classification of having a Gross National Income (GNI) of $12,696 or more [18]. Additional file 1 provides a list of all countries that were eligible for inclusion.

Exclusion criteria were papers published in any other language than English, studies from low- and middle-income countries, or studies including specific population groups such as patients (e.g., trauma survivors), students (e.g., first year nursing students), or employees from a specific setting (e.g., hospitality service workers). Moreover, studies were excluded that provided insufficient data on the mean age or age range of their study sample, as otherwise it would not be possible to determine whether the majority of their sample was of reproductive age.

### Further specification of the outcome measures

The constructs of coping and resilience capture similar adaptation processes and are often used interchangeably. Yet, some nuances should not be neglected [19]. Coping refers to cognitive or behavioural attempts to manage the effects of risk factors or stressors [20]. Coping can have either a positive or negative effect, depending on the strategies being used. Often, coping strategies are subdivided into three categories: problem-focused, emotion-focused, and avoidance coping. Problem-focused coping is considered the most adequate strategy. An example of this strategy includes problem-solving coping, which is aimed at resolving the stressor [21]. Emotion-focused coping is considered less adequate. An example of this strategy is detached coping, which is aimed at handling the emotions that are paired with a stressor, but not the stressor itself [22]. Avoidance coping is considered the least adequate strategy. This strategy is characterised by, for example, disengagement coping, in which the stressor is denied or suppressed without taking further action [21]. Resilience refers to the capacity to thrive after being faced with stressors [23, 24]. A higher level of resilience means adapting better to stressors.

### Search strategy

The electronic databases EMBASE, Medline, PsycINFO, CINAHL, Google Scholar, Web of Science, and Cochrane Library were systematically searched on February 2nd, 2018. The initial search was updated on December 2nd, 2021. The search strategy consisted of Mesh and free-text terms related to our targeted population, vulnerability (including resilience and coping), social and demographic determinants, and the study design and was tailored to each individual database (see Table 2 for the EMBASE-search strategy). There was no restriction for publication date, but a language restriction was applied to English articles only. The search was supplemented by screening reference lists of included studies.

**Table 2:** EMBASE search strategy

Search strategy EMBASE	
Block 1: outcome measures	(‘vulnerable population’/exp. OR ‘vulnerability’/de OR ‘resilience’/de OR ‘psychological resilience’/de OR ‘coping behavior’/de OR ‘adaptive behavior’/exp. OR (vulnerab* OR resilien* OR coping OR ((adaptati* OR adaptive OR adjustment*) NEAR/3 (psycholog* OR behav*))):ab,ti) AND
Block 2: social determinants	(‘social environment’/de OR ‘home environment’/de OR ‘work environment’/de OR ‘built environment’/de OR neighborhood/de OR ‘psychosocial environment’/de OR ‘life course’/de OR ‘life event’/de OR ‘life stress’/de OR ‘sociodemography’/de OR ‘socioeconomics’/de OR ‘educational status’/de OR ‘social status’/exp. OR ‘employment status’/exp. OR ‘health literacy’/de OR ‘rural area’/de OR ‘urban area’/exp. OR ‘urban population’/de OR ‘rural population’/de OR ‘urban rural difference’/de OR ‘social disadvantage’/de OR ‘social network’/de OR ((social* OR public* OR macroeconomic OR economic* OR health* OR hous*) NEAR/3 (polic*)) OR ((soci* OR cultur*) NEAR/3 (value*)) OR (((social* OR home OR psychosocial* OR living OR work OR built OR ethnic* OR cultur*) NEAR/3 (environment* OR context* OR factor* OR status* OR background OR class OR disadvantage* OR depriv* OR aspect* OR network)) OR (employment NEAR/3 status) OR unemploy* OR sociocultur* OR socio-cultur* OR neighborhood OR neighbourhood OR determinant* OR ethnic* OR cultur* OR (life NEAR/3 (course* OR event* OR transition* OR stress OR distress)) OR sociodemogra* OR socioeconomic* OR socio-economic* OR (education* NEAR/3 status*) OR (income NOT (income NEAR/3 countr*)) OR poverty OR ‘health litera*’ OR (rural NEAR/3 urban) OR ((rural OR urban OR suburban OR industr*) NEAR/3 (area* OR population* OR habitat*))):ab,ti) AND
Block 3: population and exclusions	(‘population research’/exp. OR ‘observational study’/de OR ‘cohort analysis’/de OR ‘longitudinal study’/de OR ‘prospective study’/de OR ‘retrospective study’/de OR ‘sex difference’/de OR (((population OR communit*) NEAR/3 (research* OR general OR healthy OR stud*)) OR ((observation* OR longitudinal* OR prospectiv* OR retrospecti*) NEAR/3 stud*) OR cohort* OR ((sex OR gender*) NEAR/3 differen*) OR ((male OR men OR man) NEAR/3 (women OR woman OR female) NEAR/6 differen*)):ab,ti) NOT ((juvenile/exp. OR aged/exp) NOT (adult/de OR ‘middle aged’/de OR ‘young adult’/de)) NOT ([Conference Abstract]/lim OR [Letter]/lim OR [Note]/lim OR [Editorial]/lim) AND [english]/lim NOT (‘case report’/de OR ‘case report’:ab,ti)

### Study selection, data collection, and quality assessment

Study selection and data extraction was done independently by two reviewers (alternately, LM, LSB, and LAD). Any discrepancy between the reviewers was sorted out by consulting a third reviewer (LCMB). Search results were first screened by title and abstract, thereafter full texts of eligible studies were assessed for inclusion. Relevant data from each included study was extracted using a pre-piloted data extraction form (see Additional file 2).

The Newcastle-Ottawa-Scale (NOS) was used to assess the quality of each included study [25]. This tool is suitable for assessing the quality of studies with an observational design [26, 27]. An amended version was used to score cross-sectional studies [28, 29]. The quality of studies was assessed on the domains of participants selection, e.g., whether they were representative of the average community, the comparability of cohorts, e.g., controlling for the most important confounders and mediators, and the outcome of interest, e.g., the method of measuring the outcome. Studies were classified as high quality (≥7 stars of maximum 9 stars), moderate quality (4-6 stars), or low quality (< 4 stars) [30]. This assessment was done independently by two reviewers (alternately, LM, LSB, and LAD).

### Summary measures

The main characteristics of each included study are summarised and presented in a tabulated manner, with the studies sorted by their outcome measure and the scale used to assess the outcome measure. The findings on the associations between social determinants and the outcome measure are narratively summarised, grouped per social determinant. For studies that assessed coping as the outcome measure, findings are further divided into adequate, less adequate, and inadequate coping strategies. Additional file 3 explains which coping strategy falls under which subdivision. No meta-analysis was performed due to substantial heterogeneity in the measurement of determinants and outcomes.

## Results

**Fig. 1:**
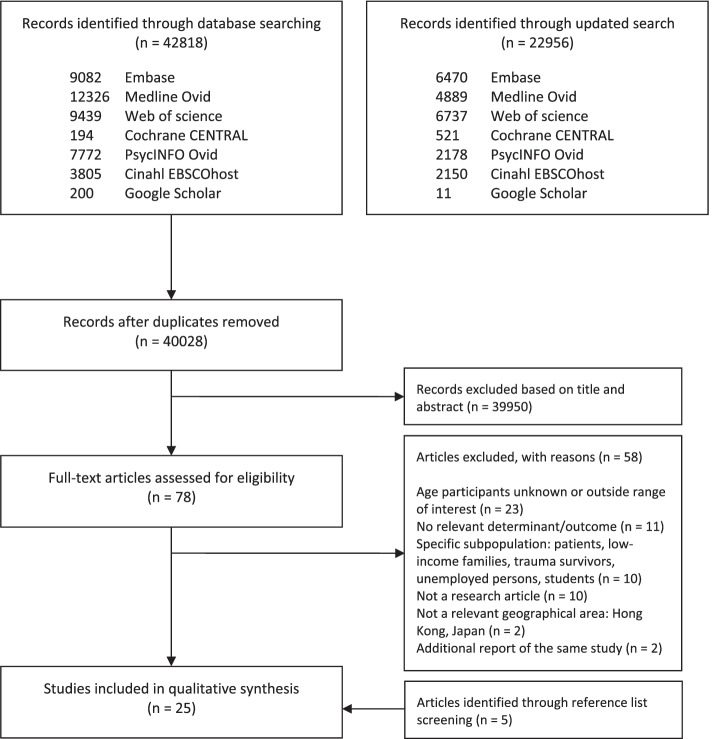
Prisma flow chart

The electronic database search yielded 65,774 citations, with 40,028 unique records after removing duplicates (Fig. 1). After title and abstract screening, 78 eligible articles remained for full text reading. Deviations in geographical area or study population were the main reasons for exclusion. After full text examination, 56 articles were excluded (reasons available in Additional file 4). An additional five articles were discovered by reference lists screening of included articles. Twenty-seven articles fulfilled our eligibility criteria. In two cases, an article turned out to be an additional report of the same study addressing the same determinants. These have been omitted from the evidence synthesis, to avoid double counting of the findings. Consequently, the final number of included articles in this review was 25.

### Description of included studies

Table 3 summarises the main characteristics of the included studies (additional characteristics in Additional file 5). Eleven studies were conducted in North America (44%), ten in Europe (40%), and three in Australia or New Zealand (12%). The years of publication ranged from 1964 to 2021. Twenty-one studies had a cross-sectional study design (84%), the other four a cohort design (16%). Of the two outcome measures, coping was most used (*n* = 17, 68%). A variety of scales was used to measure coping or resilience. A detailed summary of the used scales, along with an adequacy description of the coping strategies, is available in Additional file 3. The risk of bias assessment revealed four studies to be of low quality (20%), sixteen studies to be of moderate quality (64%), and five of high quality (20%) (see Table 4).

**Table 3:** Main characteristics of included studies

First author (year of publication)	Country^a^	Study design^b^	Sampling method (size)	Age range (mean age)^c^	Scale^d^	Risk of bias
			**Coping**			
Irion (1987) [31]	US	CS	Convenience (24)	18-25 (20.1)	WCC	3
Vingerhoets (1990) [32]	NL	CS	Convenience (997)	25-50 (M:36.4, F:34.9)	WCC	5
De Ridder (1995) [33]	NL	CS	Unspecified (261)	18-65 (37.6)	WCC	6
Alexander (2001) [34]	AU	C	Convenience (184)	- (M:30.9, F:28.7)	WCC(R)	5
Pallant (2002) [35]	AU	CS	Snowball (439)	18-82 (37.0)	COPE(B)	4
Batsikoura (2021) [36]	GR	CS	Stratified (693)	> 18 (31.7)	COPE	7
Matud (2004) [37]	ES	CS	Convenience (2816)	18-65 (M:31.9, F:34.3)	CSQ	5
Melendez (2012) [38]	ES	CS	Convenience (92)	18-39 (22.9)	CSQ	3
Cronqvist (1997) [39]	SE	CS	Stratified (45)	26-40 (−)	JCS	5
Lindqvist (2000) [40]	SE	CS	Random (91)	18-29 (−)	JCS	6
Haan (1964) [41]	US	C	Stratified (99)	37 (−)	No scale	4
Holahan (1987) [42]	US	C	Random (405)	- (39.4)	HDL	5
Anderson (1991) [43]	US	CS	Convenience (164)	22-63 (33.0)	F-COPES	3
Harnish (2000) [44]	US	CS	Stratified (763)	21-26 (23.6)	No scale	6
Roussi (2006) [45]	GR	CS	Convenience (186)	19-72 (U:37.5, R:40.4)	SACS	5
Howerton (2009) [46]	US	CS	Stratified (1784)	18-21 (−)	MCI	8
Amirkhan (2017) [47]	US	CS	Convenience (255)	18-85 (37.9)	CSI	7
			**Resilience**			
Campbell-Sills (2009) [48]	US	CS	Random (318)	18-44 (−)	CD-RISC	8
Tomyn (2018) [49]	AU	CS	Convenience (1000)	16-25 (20.8)	CD-RISC	7
Pulido-Martos (2020) [50]	ES	CS	Snowball (1011)	18-59 (32.1)	CD-RISC	6
Yu (2021) [51]	US	CS	Convenience (207)	18-75 (33.6)	CD-RISC	3
Friborg (2003) [52]	NO	C	Random (276)	25-50 (M:37.1, F:35.6)	RSA(A)	5
Capanna (2013) [53]	IT	CS	Snowball (197)	18-55 (36.1)	RSA	4
Simeon (2007) [54]	US	CS	Convenience (54)	18-60 (33.2)	DSQ	5
Montoya-Williams (2020) [55]	US	CS	Stratified (15701)	24-32 (28.4)	AHRI	6

^a^*US* United States, *NL* the Netherlands, *AU* Australia, *GR* Greece, *ES* Spain, *SE* Sweden, *NO* Norway, *IT* Italy. ^b^ C Cohort study, *CS* Cross-sectional study. ^c^*M* Males, *F* Females, *U* Urban, *R* Rural. ^d^ *WCC(−R)* Ways of Coping Checklist (Revised), (brief) *COPE* Coping Orientation of Problem Experienced, *CSQ* Coping Styles Questionnaire, *JCS* Jalowiec Coping Scale, *HDL* Health and Daily Living form, *F-COPES* Family Crisis Oriented Personal Scales, *SACS* Strategic Approach to Coping Scale, *MCI* Multidimensional Coping Inventory, *CSI* Coping Strategy Indicator, *CD-RISC* Connor-Davidson Resilience Scale, *RSA* Resilience Scale for Adults (Amended), *DSQ* Defense Style Questionnaire, *AHRI* Add Health Resilience Instrument

**Table 4:** Risk of bias (Newcastle Ottawa Scale)

Study (year of publication)	Scale^a^	Design^b^	Risk of bias components			Quality^c^	Stars
			Selection (max. 4 stars)	Comparability (max. 2 stars)	Exposure / outcome (max. 3 stars)		
			**Coping**				
Irion (1987) [31]	WCC	CS	**	–	*	Low	3
Vingerhoets (1990) [32]	WCC	CS	***	–	**	Moderate	5
De Ridder (1995) [33]	WCC	CS	***	*	**	Moderate	6
Alexander (2001) [34]	WCC(R)	C	***	–	**	Moderate	5
Pallant (2002) [35]	COPE(B)	CS	**	*	*	Moderate	4
Batsikoura (2021) [36]	COPE	CS	****	*	**	High	7
Matud (2004) [37]	CSQ	CS	****	–	*	Moderate	5
Melendez (2012) [38]	CSQ	CS	**	–	*	Low	3
Cronqvist (1997) [39]	JCS	CS	***	–	**	Moderate	5
Lindqvist (2000) [40]	JCS	CS	****	–	**	Moderate	6
Haan (1964) [41]	No scale	C	**	–	*	Low	3
Holahan (1987) [42]	HDL	C	***	*	**	Moderate	6
Anderson (1991) [43]	F-COPES	CS	**	–	*	Low	3
Harnish (2000) [44]	No scale	CS	****	–	**	Moderate	6
Roussi (2006) [45]	SACS	CS	***	–	**	Moderate	5
Howerton (2009) [46]	MCI	CS	****	**	**	High	8
Amirkhan (2017) [47]	CSI	CS	***	**	**	High	7
			**Resilience**				
Campbell-Sills (2009) [48]	CD-RISC	CS	****	**	**	High	8
Tomyn (2018) [49]	CD-RISC	CS	****	*	**	High	7
Pulido-Martos (2020)	CD-RISC	CS	****	–	**	Moderate	6
Yu (2021) [51]	CD-RISC	CS	**	–	*	Low	3
Friborg (2003) [52]	RSA(A)	C	****	–	*	Moderate	5
Capanna (2013) [53]	RSA	CS	***	–	*	Moderate	4
Simeon (2007) [54]	DSQ	CS	***	–	**	Moderate	5
Montoya-Williams (2020) [55]	AHRI	CS	****	–	**	Moderate	6

^a^*WCC(−R)* Ways of Coping Checklist (Revised), (brief) *COPE* Coping Orientation of Problem Experienced, *CSQ* Coping Styles Questionnaire, *JCS* Jalowiec Coping Scale, *HDL* Health and Daily Living form, *F-COPES* Family Crisis Oriented Personal Scales, *SACS* Strategic Approach to Coping Scale, *MCI* Multidimensional Coping Inventory, *CSI* Coping Strategy Indicator, *CD-RISC* Connor-Davidson Resilience Scale, *RSA* Resilience Scale for Adults (Amended), *DSQ* Defense Style Questionnaire, *AHRI* Add Health Resilience Instrument. ^b^*C* Cohort study, *CS* Cross-sectional study. ^c^Low quality (≤3 stars), moderate quality (4-6 stars), high quality (≥7 stars)

### Determinants

A total of 30 determinants of coping or resilience were identified (Table 5). Below, the findings are discussed per determinant, grouped into five headings: demographics, socioeconomic attainment, social environment, psychosocial well-being and life experiences, and location. An extensive summary of the findings is provided in Additional file 6.

**Table 5:** Overview of analysed determinants in included studies

Determinant	No. of times included	Reference number to study	Statistically significant association found	
			Yes	No
*Demographics*				
Age	8	**Coping:** 36, 37, 47	3	0
		**Resilience:** 48, 49, 52, 54, 55	3	2
Gender	15	**Coping:** 32, 33, 34, 36, 37, 38, 39, 40, 46, 47	9	1
		**Resilience:** 49, 50, 52, 54, 55	4	1
Ethnicity	3	**Coping:** 46	1	0
		**Resilience:** 51, 55	0	2
*Socioeconomic attainment*				
Educational level	7	**Coping:** 33, 36, 37, 42, 47	5	0
		**Resilience:** 52, 55	1	1
Income	4	**Coping:** 42, 47	2	0
		**Resilience:** 49, 55	2	0
Employment status	1	**Resilience:** 52	1	0
Employment years	1	**Resilience:** 52	1	0
Employment arrangements	1	**Coping:** 43	1	0
Socioeconomic status	2	**Coping:** 41, 46	2	0
Social mobility	1	**Coping:** 41	1	0
*Social environment*				
Marital status	1	**Coping:** 33	0	1
Household size	1	**Coping:** 37	1	0
Family support	1	**Coping:** 42	1	0
Social support	2	**Coping:** 33, 34	1	1
Sense of coherence	1	**Coping:** 35	1	0
Sense of community	1	**Coping:** 45	1	0
Social connectedness	1	**Resilience:** 53	1	0
Acculturation	1	**Resilience:** 51	1	0
*Psychosocial well-being and life experiences*				
Chronic strain/stress	2	**Coping:** 37, 46	2	0
Daily strain/stress	2	**Coping:** 34, 37	2	0
Characteristics stressors^a^	2	**Coping:** 32, 33	2	0
Domain of stressor	2	**Coping:** 31, 44	1	1
Childhood trauma	2	**Coping:** 47	1	0
		**Resilience:** 54	1	0
Lifetime trauma	1	**Coping:** 47	1	0
Life events	1	**Coping:** 42	1	0
Characteristics life events^b^	1	**Coping:** 37	1	0
Psychosocial well being	3	**Coping:** 32, 36, 37	3	0
Satisfaction with life	1	**Coping:** 33	1	0
Work role satisfaction	1	**Coping:** 37	1	0
*Location*				
Urbanisation	2	**Coping:** 36, 45	2	0

^a^Severity, unpleasantness, and manageability of stressors. ^b^Number, uncontrollability, and undesirability of life events

#### Demographics

Seventeen studies (68%) included demographic variables (age, gender, or ethnicity) as determinants. Eight studies assessed the relationship between age and coping (*n* = 3) or resilience (*n* = 5). All three coping studies reported a significant and positive association between age and adequate coping [36, 37, 47]. Three of the five resilience studies reported similar results, namely that individuals of older age groups had higher resilience scores compared to their younger counterparts [48, 49, 52]. Two resilience studies did not find a significant association [54, 55].

Fifteen studies assessed the relationship between gender and coping (*n* = 10) or resilience (*n* = 5). Four coping studies reported women using more adequate coping strategies than men did [32, 37, 40, 46]. In contrast, three other coping studies reported the opposite [33, 34, 36], one study did not find a significant association [47], and the results of two studies were inconclusive [38, 39]. Three of the five resilience studies reported higher resilience scores for men [49, 50, 55], one study reported no apparent differences between men and women [52], and one study did not find a significant association [54].

Three studies assessed the association between ethnicity and coping (*n* = 1) or resilience (*n* = 2). One study reported that individuals with a Hispanic or African American background used more inadequate coping strategies compared to Caucasian individuals [46]. The other two studies reported no significant differences between individuals of varying ethnic backgrounds [51, 55].

#### Socioeconomic attainment

Eleven studies (44%) examined the association between socioeconomic variables (educational level, income, employment, or socioeconomic status) and the outcome measures. Seven studies included educational level as determinant of coping (*n* = 5) or resilience (*n* = 2). Three of the five coping studies reported that with increasing educational level, individuals utilised more adequate coping and less inadequate coping strategies [37, 42, 47]. One coping study found the opposite effect, namely that individuals with a lower educational level showed less inadequate coping strategies [36]. In one study the findings were inconclusive [33]. One of the two resilience studies reported higher levels of resilience for individuals with a higher educational level [55]. The other study did not find a significant association [52].

Income was included as a determinant of coping (*n* = 2) or resilience (*n* = 2) in four studies. All studies found comparable results. Individuals with a higher income used less inadequate coping strategies or scored higher on the resilience scales, compared to their counterparts with a lower income [42, 47, 49, 55].

Two studies examined the association between employment-related variables and coping (*n* = 1) or resilience (*n* = 1). The coping study assessed employment arrangements between partners and found more inadequate coping for men and more adequate coping for women when both partners were employed, compared to situations where the woman worked less or stayed at home [43]. The resilience study showed a positive association between being employed as well as the number of working-years and resilience [52].

Two studies included a composite measure of SES during childhood (*n* = 1) or adulthood (*n* = 1) as a determinant of coping. Having a higher (parental) SES while growing up was associated with more adequate coping for men, but not for women, during adulthood. A higher SES during adulthood was associated with more adequate coping for both men and women [41, 46]. Furthermore, when examining social mobility by assessing the differences between SES during child- and adulthood, it was discovered that both men and women used more adequate coping strategies when their SES was higher in adulthood than during childhood [41].

#### Social environment

Eight studies (32%) examined variables related to individuals’ social environment such as their family, their network, or the community they live in. Two studies included family characteristics as determinants of coping. The first study examined the association between marital status and coping and did not find a significant association [33]. The second study examined the association between the number of children and coping and reported that having more children was associated with more use of adequate coping strategies for men, but not for women [37].

Three studies investigated social support and its effect on coping. One study reported that perceiving the amount and quality of social support from significant others as sufficient was associated with more use of adequate coping strategies [42]. One study found similar results, but only for women and not for men [34]. The other study did not find a significant effect [33].

Four studies examined the association between the community environment and coping (*n* = 2) or resilience (*n* = 2). Individuals that felt that they belonged to a close community, used more adequate coping strategies [45]. This was also true for individuals that perceived their social world as coherent. These associations were more pronounced in women compared to men [35]. Furthermore, individuals that felt connected with their social environment scored higher on the resilience scale [53]. Finally, individuals that identified and felt comfortable with the dominant culture in society scored higher on the resilience scale compared to individuals who felt this to a lesser extent [51].

#### Psychosocial well-being and life experiences

Eleven studies (44%) examined the association between stressors, life events, psychosocial well-being, and life satisfaction and the outcome measures. The findings of the relationship between chronic stressors (i.e., long lasting conflicts in one or various domains of life) or daily stressors (i.e., common demands during everyday life) and coping (*n* = 4) were ambiguous. Individuals experiencing chronic or daily stressors utilised inadequate as well as adequate coping strategies [34, 37, 46]. Four studies addressed stressor characteristics, such as the severity, manageability or domain of the stressor. Individuals that rated stressors as severe, unpleasant, as well as manageable used more adequate coping strategies [32, 33]. Individuals facing interpersonal stressors used more inadequate coping strategies than when facing stressors from other domains (for example, transition or illness-related stressors) [44]. Finally, no apparent differences were discovered between stressors that were perceived as threatening or challenging [31].

Four studies reported on the association between traumatic or adverse life events and coping (*n* = 3) or resilience (*n* = 1). Having dealt with traumatic or adverse life events, for example the experience of emotional or physical abuse or losing a loved one, during childhood or any other time in life, was associated with inadequate coping or lower resilience [47, 54]. Moreover, higher numbers of adverse events were associated with more inadequate coping for women. Feelings of uncontrollability and undesirability concerning the adverse life events were associated with using inadequate coping strategies for both men and women [37].

Four studies assessed the relationship between psychosocial well-being or satisfaction with life and coping. Two studies reported that lower psychosocial well-being, for instance when individuals experienced negative feelings or were feeling blue, was associated with more inadequate coping [32, 37]. One study reported inconclusive findings regarding the effect of psychosocial well-being [36]. Finally, the results on the effect of satisfaction with (working) life were equivocal [33, 37].

#### Location

Two studies (8%) examined place of living and its effect on coping. One study reported individuals from smaller cities using more adequate coping strategies than individuals from larger cities did [36]. The other study reported that both individuals from urban areas and rural areas used more inadequate coping strategies. However, individuals from rural areas combined those strategies more often with adequate strategies as well [45].

## Discussion

The 25 included observational studies examined a total of 30 different determinants of vulnerability, i.e., inadequate coping or reduced resilience, in the population of reproductive age. The most commonly assessed determinants were age and gender. Older individuals used more adequate coping strategies or were more resilient compared to their younger counterparts. This may be explained by the fact that when people age, they are better able to regulate their emotions. Experiencing less negative emotions enables the use of problem-solving coping strategies [56]. Men were notably more resilient than women, but gender differences in coping strategies were ambiguous. Previous studies reported similar results [57]. Gender differences appear to be context dependent, meaning that the situation to be dealt with is decisive for the strategies men and women employ [58, 59]. A lower socioeconomic attainment, with educational level as most frequently examined determinant, was associated with inadequate coping or less resilience. This effect has also been observed in other (sub)populations [60, 61]. It seems that in addition to having fewer resources, disadvantaged individuals more often have a diminished belief in control over life and are therefore less likely to adopt adequate coping strategies aimed at tackling problems [62–64]. We observed that determinants related to the social environment show a consistent effect in the same direction. Experiencing sufficient support, belonging to a close community and viewing the social world as coherent were all associated with adequate coping or greater resilience. This concurs with other studies showing that having significant others to fall back on brings on a feeling of empowerment which is a positive incentive to seek help to deal with stressors [65–68]. Contrarily, findings on experiencing daily or chronic stress were less conclusive. Stress can elicit adequate as well as inadequate coping strategies. More pronounced were the effects of negative or traumatic life events, which were associated with inadequate coping strategies. Few studies addressed the effect of the living environment (e.g., housing or neighbourhood’s green spaces). We found minor evidence for the degree of urbanisation. Individuals from urbanised areas tend to use more inadequate coping strategies than individuals living in rural areas.

Some associations between social determinants and coping or resilience appear to be influenced by sex differences. For example, having multiple children is associated with adequate coping in men, but not in women. Studies have shown that women experience less friction when reconciling family life and work than men do [69], which would suggest that men show less adequate coping strategies when having more children. Yet, other studies have shown that having more children elicits a stronger focus on having and maintaining a job in men [70]. Possibly, fatherhood induces a greater sense of responsibility, leading to more adequate coping strategies for men. In another example, social context appears to have a stronger association with coping or resilience in women than in men. This may be explained by women having higher levels of social support more often than men [71, 72]. We identified sex differences only in some studies, as not all studies included sex as a grouping variable. Therefore, it is difficult to state whether these differences are robust. However, within the medical literature it is well established that men and women are differently burdened by illness throughout their life, with a complex interplay of biological, social, and behavioural determinants that underlie these differences [73]. This leads to the expectation that the pathways from social determinants of health to health outcomes such as coping or resilience vary to some extent for women and men.

We expected to find a substantial number of studies examining socioeconomic determinants, given the evidence for their influence on health-related outcomes [2–4]. Surprisingly, less than half of the included studies investigated a socioeconomic determinant such as education, employment, or income. A possible explanation can be that SES-determinants are not often included in studies on coping and resilience. The concepts of coping and resilience have their origins in the field of psychology. It is common to focus on the influence of psychological traits on the capacity to cope with, or be resilient against, adversities [74]. While psychological traits were beyond the scope of this review, the importance of these traits on individuals’ coping or resilience abilities should not be ignored.

The findings presented in this review are mostly based on correlational studies, therefore we do not make statements about the causality of the discovered relationships. It is likely that certain social determinants and coping or resilience mutually influence or reinforce each other. Social determinants are widely understood to be interconnected; a change in one domain can bring about a change in another domain [75]. For instance, a lower educational attainment can impact job opportunities and limit income. A lower income can introduce financial troubles and stress, which can endorse the use of inadequate coping strategies, ultimately affecting health.

We used the concepts of (inadequate) coping and resilience as proxies for vulnerability. Studies targeting vulnerable groups often refrain from providing a description of vulnerability [76, 77]. However, without conceptualising vulnerability it is difficult to compare studies and synthesise evidence. When studies do conceptualise vulnerability, it is common to use a low socioeconomic position as a proxy of vulnerability. Yet, socioeconomic position and vulnerability are not interchangeable because not all individuals with a low socioeconomic position are vulnerable and vice versa [78]. Furthermore, such an approach eliminates the possibility of investigating the influences of socioeconomic variables on vulnerability. By using the concepts of coping and resilience, we have been able to bring together results from various studies and investigate the influence of socioeconomic determinants, and other social determinants, on vulnerability.

### Strengths and limitations

To our knowledge, this is the first study to provide a comprehensive summary of evidence on social determinants of vulnerability in the reproductive life stage. While it is common to target a set of pre-specified determinants when conducting a systematic review, we specifically aimed to identify all possible relevant social determinants. This approach has led to an extensive search in multiple databases and, combined with supplementary reference list screenings, enabled us to provide a unique outline of the social determinants of vulnerability that matter in this stage of life.

This review has limitations that merit discussion. There was considerable heterogeneity in the measurements of determinants and outcomes between the included studies. This hampered the possibility to pool findings, make statements about the strength of the associations, and draw strong conclusions. Another limitation concerns the assessment of whether a coping strategy is adequate or inadequate. We made use of a commonly used subdivision into problem-focused, emotion-focused, and avoidance strategies, ranging from more adequate to more inadequate, respectively. While this is a generalisation that may apply to many people, it is possible some strategies will work for some and not for others and their adequacy will depend on the stressor being addressed. Furthermore, this review reconfirmed that the general population of reproductive age is not often included in research. During the screening process, most studies were excluded because the targeted study population did not match our intended population. Of the studies that were included, many made use of a convenience sampling method. A disadvantage of convenience sampling is that not everyone has an equal opportunity to participate in the study, which can lead to underrepresentation of particularly harder to reach individuals [79]. The study populations of the included studies may not have been an accurate reflection of the actual general population during this life stage. Lastly, the characteristics of the included studies could have formed a limitation. Our search strategy was restricted to studies published in English, leaving possible relevant studies in other languages undiscovered. Furthermore, we included studies that were conducted in high-income countries in Europe, North-America, Australia, and New Zealand, as these countries have comparable demographic characteristics [80]. Not considering studies from other high-income countries, as well as low- and middle-income countries, may have led to evidence on the association between social determinants and coping or resilience being undiscovered. Lastly, most of the included observational studies were rated as moderate in quality, making the findings less reliable.

### Implications for research, practice, and policy

Our review can serve as a starting point for further research concerning vulnerability in the general population of reproductive age, including all parents and prospective parents. Several research gaps became apparent, such as the scarcity of research on vulnerability in this group or the inclusion of social determinants in research on vulnerability. More high-quality research should be conducted to strengthen the evidence on social determinants of vulnerability and gain insight into the (relative) effects of each determinant. This is especially relevant as there is an increased focus on health promotion before pregnancy, to optimise the health of the next generation as early as possible [81, 82]. Moreover, research on the social determinants of coping or resilience in other settings, such as in low- or middle-income countries, could provide further understanding about the role of social determinants. It is unclear whether social determinants are similarly associated with coping and resilience in these settings, or whether different mechanisms are at play. Additionally, we recommend future studies to include sex as a grouping variable in their analysis to gain more insight into the possible different associations between social determinants and vulnerability for men and women. Finally, it is advisable to use more uniform definitions and measurements of vulnerability, including coping and resilience, to reach comparability between different studies.

This first overview of relevant social determinants of vulnerability during the reproductive life stage can be used to better identify possibly vulnerable (prospective) parents and to develop tailored interventions aimed at reducing vulnerability in this group. It emphasises that, additional to one’s socioeconomic position, other social determinants related to the social environment, living environment or psychological well-being must not be overlooked. Moreover, the understanding of how social determinants and vulnerability are associated differently in men and women, might enable the development of tailored interventions aimed at maximising the effectiveness and individuals’ engagement. Furthermore, the implications of knowledge about social determinants of vulnerability in the general population aged 18-40 years may even reach further than the subject of achieving healthy pregnancies and optimizing the health of unborn or new-born children. For example, reducing vulnerability may help to increase labour market participation or work productivity. Also, insights in the factors that contribute to vulnerability may help to optimise the provision of health care.

## Conclusions

Our findings show that a variety of social determinants are related to inadequate coping or low resilience in the general population of reproductive age, including a younger age, a low socioeconomic attainment, lack of social support, incoherent view of the social world, as well as adverse life events. Taking these social determinants into consideration can contribute to the better identification of vulnerable individuals and to the development of tailored interventions to reduce vulnerability in this group. However, more high-quality research on social determinants of vulnerability in the population of reproductive age must be conducted.

## Supplementary Information

**Additional file 1.** High-income countries. List of high-income countries as specified by the World Bank Organization.

**Additional file 2.** Data extraction form. List of topics on which data has been collected from the included studies.

**Additional file 3.** Coping and resilience scales. Used coping and resilience scales by the included studies, alongside with adequacy description of different coping or resilience strategies.

**Additional file 4.** Reasons for excluding potential eligible studies. Overview of reasons for the exclusion of potential eligible studies during title and abstract screening.

**Additional file 5.** Main characteristics of included studies (extended). Elaborated table with main characteristics of the included studies.

**Additional file 6.** Extended narrative results. Elaborated table with all important narrative findings of the included studies.

## Data Availability

Data are available through corresponding author.

## Abbreviations

SES: Socioeconomic status
WHO: World Health Organization
SDOH: Social determinants of health
NOS: Newcastle-Ottawa Scale
