# Supplementary material for: Social determinants of vulnerability in the population of reproductive age: a systematic review
Source: BMC Public Health. 2022 Jun 24;22:1252. doi: 10.1186/s12889-022-13651-6 (PMC9233331; doi:10.1186/s12889-022-13651-6)
Supplement: Supplementary file 1 — Additional file 1. High-income countries. List of high-income countries as specified by the World Bank Organization. [file 12889_2022_13651_MOESM1_ESM.docx]

**Additional file 1. List of high-income countries eligible for inclusion, sorted in alphabetical order.**

Countries in Europe, North-America, Australia and New Zealand with a Gross National Income (GNI) of $12,696 or higher, as defined by the World Bank Country in 2021.

Andorra

Australia

Austria

Belgium

Canada

Croatia

Cyprus

Czech Republic

Denmark

Finland

France

Germany

Greenland

Greece

Hungary

Ireland

Italy

Liechtenstein

Lithuania

Luxembourg

Monaco

Netherlands

New Zealand

Norway

Poland

Portugal

Romania

Sweden

Switzerland

United Kingdom

United States
