# Supplementary material for: Social determinants of vulnerability in the population of reproductive age: a systematic review
Source: BMC Public Health. 2022 Jun 24;22:1252. doi: 10.1186/s12889-022-13651-6 (PMC9233331; doi:10.1186/s12889-022-13651-6)
Supplement: Supplementary file 2 — Additional file 2. Data extraction form. List of topics on which data has been collected from the included studies. [file 12889_2022_13651_MOESM2_ESM.docx]

**Additional file 2. Data extraction form.**

| Theme | Topics |
| --- | --- |
| Study characteristics | First author  Year of publication  Country/setting  Study aim  Study period  Study design |
| Population characteristics | Sampling method  In- and exclusion criteria  Number of participants  Population description |
| Determinants | Determinants  Definition of determinant  Measurement of determinant  Questionnaire/scale |
| Outcomes | Outcomes  Definition of outcome  Measurement of outcome  Questionnaire/scale |
| Analysis/findings | Type of analysis  OR/RR  Narrative results  Crude results  Adjusted results  Mean rates |
| Conclusion | Strengths and limitations  Key conclusions  Risk of bias  Inclusion/exclusion with reasons  Additional notes |
