# Supplementary material for: Social determinants of vulnerability in the population of reproductive age: a systematic review
Source: BMC Public Health. 2022 Jun 24;22:1252. doi: 10.1186/s12889-022-13651-6 (PMC9233331; doi:10.1186/s12889-022-13651-6)
Supplement: Supplementary file 3 — Additional file 3. Coping and resilience scales. Used coping and resilience scales by the included studies, alongside with adequacy description of different coping or resilience strategies. [file 12889_2022_13651_MOESM3_ESM.docx]

**Additional file 3. Coping and resilience scales.**

| Scale | Subscales/strategies | Adequacy assessment |
| --- | --- | --- |
| Coping | | |
| **Coping Strategy Indicator (CSI)** | Problem-solving  Seeking social support  Avoidance | Adequate^1^  Adequate^2^  Inadequate^1 3^ |
| **Jalowiec Coping Scale (JCS-60)** | Confrontive  Evasive  Optimistic  Fatalistic  Emotive  Palliative  Supportant  Self-reliant | Adequate^4^  Adequate^4^  Adequate^4^  Inadequate^4^  Less adequate^4^  Less adequate^4^  Adequate^4^  Adequate^4^ |
| **Coping Orientation to Problems Experienced (COPE)** | Active coping  Planning  Suppression of competing activities  Restraint  Use of instrumental social support  Use of emotional social support  Positive reinterpretation and growth  Acceptance  Denial  Religious coping  Focus on and venting of emotions  Humor  Behavioral disengagement  Mental disengagement  Substance use | Adequate^5-8^  Adequate^5-8^  Adequate^6 7^  Less adequate^6 7^  Adequate^6 7^  Adequate^6 7^  Less adequate^6 7^  Less adequate^6 7^  Inadequate^6-8^  Less adequate^6 7^  Adequate^6 7^  Inadequate^6 7^  Inadequate^6 7^  Inadequate^6-8^  Inadequate^6 7^ |
| **Health and Daily Living Form (HDL)** | Active-cognitive  Active-behavioral  Avoidance-oriented | Adequate^9^  Adequate^9^  Inadequate^9^ |
| **Coping Styles Questionnaire (CSQ)** | Rational coping  Detached coping  Emotional coping  Avoidance coping | Adequate^10 11^  Less adequate^10^  Less adequate^10 11^  Inadequate^10^ |
| **Ways of Coping Checklist (WCC)** | Confrontive coping  Distancing  Self-controlling  Seeking social support  Accepting responsibility  Escape-Avoidance  Planful problem-solving  Positive reappraisal | Adequate^21 22^  Inadequate^21 22^  Less adequate^21 22^  Adequate^21 22^  Less adequate^21 22^  Inadequate^21 22^  Adequate^21 22^  Adequate^21 22^ |
| **Strategic Approach to Coping (SACS)** | Assertive action  Cautious action  Social joining  Seeking social support  Antisocial action  Aggressive action  Indirect action  Avoidance  Instinctive action | Inadequate^12 13^  Adequate^12 13^  Adequate^12 13^  Adequate^12 13^  Less adequate^12 13^  Less adequate^12 13^  Inadequate^12 13^  Inadequate^12 13^  Less adequate^12 13^ |
| **Family Crisis Oriented Personal Scales (F-COPES)** | Acquiring social support  Reframing the problem to make it more manageable  Seeking spiritual support  Mobilizing the family to acquire professional or community help  Passively accepting the problem | Adequate^14^  Adequate^14^  Adequate^14^  Adequate^14^  Adequate^14^ |
| **Multidimensional Coping Instrument (MCI)** | Task-oriented  Emotion-oriented  Avoidance-oriented | Adequate^15 16^  Less adequate^15 16^  Inadequate^15 16^ |
| Resilience | | |
| **Resilience Scale for Adults (RSA)** | Perception of self  Planned future  Social competence  Structured style  Family cohesion  Social resources | Higher scores resemble higher resilience^17^ |
| **Defense Style Questionnaire (DSQ)** | Mature  Neurotic  Immature | Resilience^18^  Less resilience^18^  Less resilience^18^ |
| **Connor-Davidson Resilience Scale (CD-RISC)** | Personal competence  Acceptance of change and secure relationships  Trust/tolerance/strengthening effects of stress  Control  Spiritual influences | Higher scores resemble higher resilience^19^ |
| **Add Health Resilience Indicator (AHRI)** |  | Higher scores resemble higher resilience^20^ |

^1^Amirkhan JH, Marckwordt M. Past trauma and current stress and coping: Toward a general model. *Journal of Loss and Trauma* 2017;22(1):47-60.

^2^Felsten G. Gender and coping: Use of distinct strategies and associations with stress and depression. *Anxiety, Stress & Coping: An International Journal* 1998;11(4):289-309.

^3^Leitenberg H, Gibson LE, Novy PL. Individual differences among undergraduate women in methods of coping with stressful events: The impact of cumulative childhood stressors and abuse. *Child abuse & neglect.* 2004;28(2):181-92.

^4^Waltz, C. Measurement of Nursing Outcomes, Volume 3: Self Care and Coping. Springer Publishing Company, 2001.

^5^Melendez JC, Mayordomo T, Sancho P, et al. Coping strategies: gender differences and development throughout life span. *Span J Psychol* 2012;15(3):1089-98.

^6^Sica, Claudio, et al. "Coping strategies: Evidence for cross-cultural differences? A preliminary study with the Italian version of coping orientations to problems experienced (COPE)." Personality and individual differences 23.6 (1997): 1025-1029.

^7^Sica C, Latzman RD, Caudek C, et al. Facing distress in Coronavirus era: The role of maladaptive personality traits and coping strategies. Personality and Individual Differences. 2021 Jul;177:110833. DOI: 10.1016/j.paid.2021.110833. PMID: 34776570; PMCID: PMC8570824.

^8^Eaton, Rebecca J., and Graham Bradley. "The role of gender and negative affectivity in stressor appraisal and coping selection." International Journal of Stress Management 15.1 (2008): 94.

^9^Holahan, Charles J., and Rudolf H. Moos. "Personal and contextual determinants of coping strategies." Journal of personality and social psychology 52.5 (1987): 946.

^10^Elklit, Ask. "Coping styles questionnaire: A contribution to the validation of a scale for measuring coping strategies." Personality and Individual differences 21.5 (1996): 809-812.

^11^Matud, M. Pilar. "Gender differences in stress and coping styles." Personality and individual differences 37.7 (2004): 1401-1415

^12^Chwaszcz, Joanna, et al. "The Polish Adaptation of the Strategic Approach to Coping Scale." Roczniki Psychologiczne 23.1 (2020): 23-43.

^13^Roussi, Pagona, and Eleni Vassilaki. "The applicability of the multiaxial model of coping to a Greek population." Anxiety, Stress and Coping 14.2 (2001): 125-147.

^14^Anderson EA, Leslie LA. Coping with employment and family stress: Employment arrangement and gender differences. *Sex Roles.* 1991;24(3):223-37.

^15^Endler, Norman S., and James D. Parker. "Multidimensional assessment of coping: a critical evaluation." Journal of personality and social psychology 58.5 (1990): 844.

^16^Howerton, Amanda, and Karen Van Gundy. "Sex differences in coping styles and implications for depressed mood." International Journal of Stress Management 16.4 (2009): 333.

^17^Friborg, Oddgeir, et al. "A new rating scale for adult resilience: what are the central protective resources behind healthy adjustment?." International journal of methods in psychiatric research 12.2 (2003): 65-76.

^18^Simeon, Daphne, et al. "Factors associated with resilience in healthy adults." Psychoneuroendocrinology 32.8-10 (2007): 1149-1152.

^19^Burns, R. A., and K. J. Anstey. "The Connor–Davidson Resilience Scale (CD-RISC): Testing the invariance of a uni-dimensional resilience measure that is independent of positive and negative affect." Personality and Individual Differences 48.5 (2010): 527-531.

^20^Montoya-Williams, Diana, Molly Passarella, and Scott A. Lorch. "Retrospective development of a novel resilience indicator using existing cohort data: The adolescent to adult health resilience instrument." Plos one 15.12 (2020): e0243564.

^21^Ahlström, Gerd, and Stig Wenneberg. "Coping with illness‐related problems in persons with progressive muscular diseases: the Swedish version of the Ways of Coping Questionnaire." Scandinavian Journal of Caring Sciences 16.4 (2002): 368-375.

^22^ Ridder, DTD De. "Social status and coping: An exploration of the mediating role of beliefs." Anxiety, stress, and coping 8.4 (1995): 311-324.
