# Supplementary material for: Social determinants of vulnerability in the population of reproductive age: a systematic review
Source: BMC Public Health. 2022 Jun 24;22:1252. doi: 10.1186/s12889-022-13651-6 (PMC9233331; doi:10.1186/s12889-022-13651-6)
Supplement: Supplementary file 4 — Additional file 4. Reasons for excluding potential eligible studies. Overview of reasons for the exclusion of potential eligible studies during title and abstract screening. [file 12889_2022_13651_MOESM4_ESM.docx]

**Additional file 4. Reasons for excluding potential eligible studies.**

| **Study/first author (year of publication)** | Reason for exclusion |
| --- | --- |
| **Aldwin (1996)** | Mean age of participants too high |
| **Alonso-Tapia (2016)** | No relevant determinants, specific subpopulation |
| **Billings (1981)** | Mean age of participants too high |
| **Brantley (2002)** | Specific subpopulation |
| **Cheng (2003)** | Deviant geographical area |
| **Cicchetti (2010)** | Review of literature |
| **Connor (2006)** | Review of literature |
| **Crane (2016)** | Mean age of participants too high |
| **Creed (2006)** | Specific subpopulation |
| **Cwikel (1988)** | No relevant outcome |
| **Diehl (1996)** | Mean age of participants too high |
| **Diehl (2014)** | Mean age of participants too high |
| **Dunkley (2014)** | Mean age of participants too high, no relevant determinants |
| **Eaton (2008)** | Specific subpopulation |
| **Fallon (2019)** | Review or literature |
| **Folkman (1980)** | Mean age of participants too high |
| **Garrido-Hernansaiz (2020)** | No information on mean age of participants |
| **Hamarat (2001)** | Mean age of participants too high |
| **Hansford (2021)** | Specific subpopulation |
| **Harter (2000)** | Specific subpopulation |
| **Higgings (2010)** | Mean age of participants too high |
| **Hilpert (2016)** | No relevant outcome |
| **Holahan (1985)** | Mean age of participants too high, no relevant outcome |
| **Holahan (1987)** | Additional report of already included study |
| **Kessler (1984)** | No relevant outcome |
| **Labouvie-Vief (1987)** | No relevant outcome |
| **Lindberg (2020)** | Mean age of participants too high, no relevant outcome |
| **Lipinska-Grobelny (2011)** | Mean age of participants too high |
| **Logan-Greene (2014)** | No relevant outcome |
| **Lyons (2016)** | Mean age of participants too high |
| **Maltby (2007)** | No relevant outcome |
| **Marotz-Baden (1986)** | No information on age of participants |
| **Matheson (2005)** | Specific subpopulation |
| **McCrae (1989)** | No information on mean age of participants |
| **McDonald (2020)** | Specific subpopulation |
| **Meng (2016)** | No information on mean age of participants |
| **Meyer (1995)** | Review of literature |
| **Okvat (2011)** | Review of literature |
| **Pearlin (1978)** | No information on mean age participants |
| **Porter (1995)** | Mean age of participants too high |
| **Porter (2000)** | Mean age of participants too high |
| **Prud’Homme (2005)** | No relevant determinants |
| **Ptacek (1994)** | Specific subpopulation |
| **Roisman (2005)** | Review of literature |
| **Santiago (2012)** | Specific subpopulation |
| **Seiffge-Krenke (2006)** | No relevant determinants |
| **Short (1997)** | Mean age of participants too high |
| **Shimanoe (2018)** | Deviant geographic area |
| **Surtees (2006)** | Mean age of participants too high |
| **Terry (1989)** | No relevant outcome |
| **Trocki (1994)** | Review of literature |
| **Trouillet (2009)** | Mean age of participants too high |
| **Vingerhoets (1989)** | Additional report of already included study |
| **Wilhelm (2007)** | No relevant determinants |
| **Woodhead (2014)** | Mean age of participants too high |
| **Zakowski (2001)** | Specific subpopulation |
