# Supplementary material for: Social determinants of vulnerability in the population of reproductive age: a systematic review
Source: BMC Public Health. 2022 Jun 24;22:1252. doi: 10.1186/s12889-022-13651-6 (PMC9233331; doi:10.1186/s12889-022-13651-6)
Supplement: Supplementary file 5 — Additional file 5. Main characteristics of included studies (extended). Elaborated table with main characteristics of the included studies. [file 12889_2022_13651_MOESM5_ESM.docx]

**Additional file 5. Main characteristics of included studies (extended).**

| First author (year of publication) | Country | Study design^a^ | Aim of study | Population | Sampling method | Sample size | Age range  (mean age)^b^ | Scale^c^ | Definition of outcome | Determinants of interest | Risk of bias,  (9/NOS) |
| --- | --- | --- | --- | --- | --- | --- | --- | --- | --- | --- | --- |
| Coping | | | | | | | | | | | |
| Irion  (1987) | United States | CS | To compare coping behaviors in adulthood. | General | Convenience sampling | 24 (of 96 total) | 18-25  (20.1) | WCC | Not provided | Domain of stressor | 3 |
| Vingerhoets  (1990) | The Netherlands | CS | To compare both sexes for the scores on coping. | General | Convenience sampling | 997 | 25-50  (M:36.4, F:34.9) | WCC | Not provided | Gender, daily strain/stress, symptoms | 5 |
| De Ridder  (1995) | The Netherlands | CS | To explore the link between social status and coping, and mediational effects of stress beliefs. | General | Not specified | 261 | 18-65  (37.6) | WCC | Not provided | Educational level, perceived social support, satisfaction with life | 6 |
| Alexander  (2001) | Australia | C | To examine relations between adult attachment styles, coping resources, appraised strain and coping strategies. | General (expecting parents) | Convenience sampling | 184 | - (M:30.9, F:28.7) | WCC-R | Yes | Perceived social support, daily (parenting) strain/stress | 5 |
| Pallant  (2002) | Australia | CS | The relationship between sense of coherence and the utilization of coping strategies. | General | Snowball sampling | 439 | 18-82  (37.0) | COPE-brief | Not provided | Sense of coherence | 4 |
| Batsikoura  (2021) | Greece | CS | To investigate the relationship between nutritional habits, lifestyle, anxiety, and coping strategies. | General | Stratified sampling | 693 | >18  (31.7) | COPE | Not provided | Age, gender, educational level, urbanization | 7 |
| Matud  (2004) | Spain | CS | To explore gender differences in coping styles. | General | Convenience sampling | 2816 | 18-65  (M:31.88, F:34.3) | CSQ | Yes | Age, gender, educational level, household size (number of children), characteristics of life-events, chronic strain/stress, daily strain/stress, work role satisfaction, psychological distress | 5 |
| Melendez  (2012) | Spain | CS | To test if gender differences in coping will become more pronounced during old age. | General | Convenience sampling | 92 | 18-39  (22.9) | CSQ | Yes | Gender | 3 |
| Cronqvist  (1997) | Sweden | CS | To obtain reference values for the Jalowiec Coping Scale and assess which coping strategies are used in a random Swedish sample. | General | Stratified sampling | 45 (of 268 total) | 26-40 | JCS | Yes | Gender | 5 |
| Lindqvist  (2000) | Sweden | CS | To explore to what extent coping strategies are employed, by various demographic subgroups that deal with stressful events. | General | Random sampling | 91 (of 519 total) | 18-29  (-) | JCS | Yes | Gender | 6 |
| Haan  (1964) | United States | C | To examine the relationships of childhood and adult social status and personality with ego functioning. | General | Stratified sampling | 99 | 37  (-) | Questionnaire | Not provided | Socioeconomic status | 4 |
| Holahan  (1987) | United States | C | To identify personal and contextual determinants of three types of coping measures. | General | Random sampling | 405 | -  (39.4) | HDL | Yes | Educational level, family income, negative life events, family support | 5 |
| Anderson  (1991) | United States | CS | To explore how women and men in various employment arrangement experience and cope with stress. | General (couples) | Convenience sampling | 164 | 22-63  (33.0) | F-COPES | Yes | Gender, employment arrangement | 3 |
| Harnish  (2000) | United States | CS | To examine the relative frequency of use of specific coping strategies among those experiencing different types of stressors. | General | Stratified sampling | 763 | 21-26  (23.6) | Questionnaire | Yes | Type of stressor | 6 |
| Roussi  (2006) | Greece | CS | To see how coping responses cluster in urban and rural areas. | General | Convenience sampling | 186 | 19-72  (U:37.5, R:40.4) | SACS | Not provided | Sense of community | 5 |
| Howerton  (2009) | United States | CS | To examine sex differences in coping. | General | Stratified sampling | 1784 | 18-21  (-) | MCI | Yes | Gender, ethnicity, parental SES, chronic strain/stress | 8 |
| Amirkhan  (2017) | United States | CS | To study the correlation between trauma and coping. | General | Convenience sampling | 255 | 18-85  (37.9) | CSI | Not provided | Age, gender, educational level, income, childhood trauma, lifetime trauma | 7 |
| Resilience | | | | | | | | | | | |
| Campbell-Sills  (2009) | United States | CS | To examine resilience in relation to demographic characteristics. | General | Random sampling | 318 (of 764 total) | 18-44  (-) | CD-RISC | Yes | Age | 8 |
| Tomyn  (2018) | Australia | CS | To validate a resilience scale in a sample of young people and investigate differences according to age, gender and household income. | General | Convenience sampling | 1000 | 16-25  (20.8) | CD-RISC | Yes | Age, gender, income | 7 |
| Pulido-Martos  (2020) | Spain | CS | To analyze gender differences in resilience at the latent factor mean level. | General | Snowball sampling | 1011 | 18-59  (32.1) | CD-RISC | Yes | Gender | 6 |
| Yu  (2021) | United States | CS | To test a potential mediational effect of acculturation between ethnicity and resilience. | General | Convenience sampling | 207 | 18-75  (33.6) | CD-RISC | Yes | Ethnicity, acculturation | 3 |
| Friborg  (2003) | Norway | C | To validate a resilience scale and to assess differences between variables known to differentiate groups regarding resilience. | General | Random sampling | 276 | 25-50  (M: 37.1, F:35.6) | RSA Amended | Yes | Age, gender | 5 |
| Capanna  (2013) | Italy | CS | To test the existence of differences due to gender, age and education in resilience. | General | Snowball sampling | 197 | 18-55  (36.1) | RSA | Yes | Social connectedness | 4 |
| Simeon  (2007) | United States | CS | To explore the relationships between resilience and several factors that have been implicated in healthy adaptation. | General | Convenience sampling | 54 | 18-60  (33.2) | DSQ | Yes | Age, gender, childhood trauma | 5 |
| Montoya-Williams  (2020) | United States | CS | To create and validate a resilience indicator. | General | Stratified sampling | 15701 | 24-32  (28.4) | AHRI | Yes | Age, gender, ethnicity, educational level, income | 6 |

^a^C: Cohort study, CS: Cross-sectional study. ^b^M: Males, F: Females, U: Urban, R: Rural. ^c^WCC(-R): Ways of Coping Checklist (Revised), COPE: Coping Orientation of Problem Experienced (Brief), CSI: Coping Strategy Indicator, CSQ: Coping Styles Questionnaire, SACS: Strategic Approach to Coping Scale, CISS: Coping Inventory for Stressful Situation, MCI: Multidimensional Coping Inventory, HDL: Health and Daily Living form, F-COPES: Family Crisis Oriented Personal Scales, SOC-13: Sense of Coherence scale, SCOPE: Survey Of Coping Profile Endorsement, RSA: Resilience Scale for Adults (Amended), CD-RISC: Connor-Davidson Resilience Scale, DSQ: Defense Style Questionnaire, RS-14: Resilience Scale, AHRI: Add Health Resilience Instrument.
