# Supplementary material for: Social determinants of vulnerability in the population of reproductive age: a systematic review
Source: BMC Public Health. 2022 Jun 24;22:1252. doi: 10.1186/s12889-022-13651-6 (PMC9233331; doi:10.1186/s12889-022-13651-6)
Supplement: Supplementary file 6 — Additional file 6. Extended narrative results. Elaborated table with all important narrative findings of the included studies. [file 12889_2022_13651_MOESM6_ESM.docx]

**Additional file 6. Extended narrative results.**

| Study  (year of publication) | Determinant | Outcome  (scale) | Summary of findings | Controlled for/stratified by |
| --- | --- | --- | --- | --- |
| Demographics | | | | |
| Friborg  (2003) | Age | Resilience  (RSA) | Aging was positively associated with resilience (subscale of personal structure, r = 0.17). | n.a |
| Matud  (2004) | Age | Coping  (CSQ) | Both men and women made more use of adequate coping when they aged (subscale of rational coping, r = 0.18 and 0.11). Women tend to use also more inadequate coping when they age (subscales of detached coping, r = 0.08 and avoidance coping, r = 0.14). | Stratified by gender |
| Simeon  (2007) | Age | Resilience  (DSQ) | No significant association was discovered between age and resilience. | n.a. |
| Campbell-Sills  (2009) | Age | Resilience  (CD-RISC) | Younger participants of the age group 18 to 24 had lower resilience scores compared to older participants of the age group 25 to 34 (mean score of 29.48 versus 32.33, respectively, range of 9-40). | n.a. |
| Amirkhan  (2017) | Age | Coping  (CSI) | Aging was negatively associated with inadequate coping (subscale of avoidance strategies, r = -0.20). | n.a. |
| Tomyn  (2018) | Age | Resilience  (CD-RISC) | Participants aged 18-21 years and 22-25 years reported higher resilience scores than those aged 16-17 years (mean scores of 75.87, 76.38, and 71.51, respectively, on a scale of 0-100%). | n.a. |
| Montoya-Williams  (2020) | Age | Resilience  (AHRI) | No significant differences were found in mean resilience scores for participants under 30 of age versus participants over 30 years of age. | n.a. |
| Batsikoura  (2021) | Age | Coping  (brief-COPE) | Aging was negatively associated with inadequate coping (subscale of substance use, r = -0.01) and positively associated with using adequate coping (subscale of acceptance/planning, r = 0.02). | Gender, educational level, urbanization, lifestyle, anxiety |
| Vingerhoets  (1990) | Gender | Coping  (WCC) | Men made more use of adequate coping compared to women (subscale of problem-focused coping, mean score of 90.96 versus 88.82, range of 24-144). Women made more use of inadequate coping compared to men (subscale of emotion-focused coping, mean score of 131.63 versus 125.97, range of 60-240). | n.a. |
| De Ridder  (1995) | Gender | Coping  (WCC) | Gender was positively associated with adequate coping (subscales of seeking social support, r = 0.27 and accepting responsibility coping, r = 0.21). Women made more use of these strategies. | n.a. |
| Cronqvist  (1997) | Gender | Coping  (JCS) | Men made more use of adequate coping compared to women (subscales of confrontative coping, mean score of 2.29 versus 2.20, range of 0-3, and self-reliant coping, mean score of 1.91 versus 1.69, range of 0-3). Contrarily, women made more use of other adequate strategies compared to men (subscale of supportant coping, mean score of 1.51 versus 1.07, range of 0-3). | n.a. |
| Lindqvist  (2000) | Gender | Coping  (JCS) | Women made more use of adequate coping as well as inadequate coping compared to men (adequate coping: subscales of confrontative coping, mean score of 1.73 versus 1.46, range of 0-3, supportant coping, mean score of 1.43 versus 0.90, range of 0-3, optimistic coping, mean score of 1.82 versus 1.45, range of 0-3, and evasive coping, mean score of 1.28 versus 1.01, range of 0-3). Less or Inadequate coping: subscales of fatalistic coping, mean score of 1.34 versus 1.09, range of 0-3 and palliative coping, mean score of 1.04 versus 0.79, range of 0-3). | n.a. |
| Alexander  (2001) | Gender | Coping  (WCC-R) | Women reported more use of adequate coping compared to men (subscales of problem-focused coping, mean score of 15.17 versus 12.37, range of 0-36, and seeking support coping, mean score of 9.40 versus 5.31, range of 0-18). | n.a. |
| Friborg  (2003) | Gender | Resilience  (RSA-A) | Gender was associated with two resilience subscales. Women reported higher levels of the subscale social support (5.91 versus 5.45, range 1-7) and lower levels of the subscale personal competence (5.28 versus 5.51, range 1-7) than men did. | n.a. |
| Matud  (2004) | Gender | Coping  (CSQ) | Women made more use of inadequate coping compared to men (subscales of emotional coping, mean score of 13.19 versus 11.55, range of 1-25) and avoidance coping, mean score of 10.09 versus 9.05, range of 3-25). Men made more use of adequate coping (subscale of rational coping, mean scores of 25.06 versus 23.15, range of 14-47) as well as inadequate coping (subscale of detachment coping, mean scores of 11.61 versus 10.61, range of 1-28). | n.a. |
| Simeon  (2007) | Gender | Resilience  (DSQ) | No significant association was discovered between gender and resilience. | n.a. |
| Howerton  (2009) | Gender | Coping  (MCI) | Men used more adequate coping compared to women (subscale of problem-focused coping, mean score of 12.5 versus 12.3, range of 4-16). Women used more inadequate coping compared to men (subscales of emotion-focused, mean score of 14.4 versus 12.9, range 5-20, and avoidance coping, mean score of 5.5 versus 4.8, range of 3-12). While controlling for ethnicity and SES, being female was associated with inadequate coping (subscale of emotion-focused coping (b = 1.56) and avoidance coping (b = 0.65)). When taking the experience of chronic strain into account, the association of gender with inadequate coping decreased but remained significant (respectively, b = 1.53 and 0.54). | Ethnicity, SES, chronic strain |
| Melendez  (2012) | Gender | Coping  (CSQ) | Women made more use of inadequate coping compared to men (subscale of negative self-focus, mean score of 1.52 versus 1.45, range of 0-6) and adequate coping (subscale of seeking support, mean score of 3.06 versus 2.28, range of 0-6). Contrarily, men made more use of other inadequate coping strategies (subscale of avoidance, mean score of 2.34 versus 2.24, range of 0-6, and religion, mean score of 0.32 versus 0.30, range of 0-6). | n.a. |
| Amirkhan  (2017) | Gender | Coping  (CSI) | No significant associations were discovered between gender and coping. | n.a. |
| Tomyn  (2018) | Gender | Resilience  (CD-RISC) | Male participants reported higher mean resilience scores compared to females (77.58 versus 73.56, on a scale of 0-100%). | n.a. |
| Montoya-Williams  (2020) | Gender | Resilience  (AHRI) | Male participants scored higher mean resilience scores compared to female participants (14.6 versus 14.1, range of 0-29). | n.a. |
| Pulido-Martos  (2020) | Gender | Resilience  (CD-RISC) | In general, men had higher resilience scores compared to women (women scoring 0.09 units lower than men). | n.a. |
| Batsikoura  (2021) | Gender | Coping  (COPE) | Being female was positively associated with using adequate coping strategies (subscale of emotional/instrumental support, r = 0.28) and negatively associated with using inadequate coping strategies (subscale of substance use, r = -0.37). | Age, educational level, urbanization, lifestyle and anxiety |
| Howerton  (2009) | Ethnicity | Coping  (MCI) | Having a Hispanic or African American background compared to the Caucasian respondents was positively associated with inadequate coping (subscale of avoidance coping, b = 0.34 and 1.07, respectively), while controlling for gender and SES. The effect remained when additionally controlling for experienced chronic strain (b = 0.25 and 0.95). | Gender, SES, chronic strain |
| Montoya-Williams  (2020) | Ethnicity | Resilience  (AHRI) | No significant differences were found in mean resilience scores between participants of different ethnic backgrounds. | n.a. |
| Yu  (2021) | Ethnicity | Resilience  (CD-RISC) | No differences were found between participants of different ethnical background when comparing resilience scores. | n.a. |
| Socioeconomic attainment | | | | |
| Holahan  (1987) | Educational level | Coping  (HDL) | Educational level was positively associated with adequate coping at time 1 (subscale of active-behavioral coping, r = 0.12) and at time 2 (subscales of active-behavioral coping, r = 0.15 and active-cognitive coping, r = 0.08). At time 2, after controlling for initial coping at time 1, educational level remained positively associated with adequate coping (subscale of active-behavioral coping, r = 0.11). Educational level was negatively associated with inadequate coping at time 1 and 2 (subscale of avoidance coping, r = -0.17 and -0.11). At time 2, when controlling for initial coping at time 1, this association disappeared. | Initial coping |
| De Ridder  (1995) | Educational level | Coping  (WCC) | Educational level was positively as well as negatively associated with adequate coping strategies (subscales of seeking social support coping, r = 0.24, and subscale of threat minimization coping, r = -0.17). | n.a. |
| Friborg  (2003) | Educational level | Resilience  (RSA) | No significant associations were discovered between years of completed education and resilience subscales. | n.a. |
| Matud  (2004) | Educational level | Coping  (CSQ) | Educational level was negatively associated with inadequate coping for women (subscales of emotional coping, r = -0.12, detachment coping, r = -0.10, and avoidance coping, r = -0.21) and for men (subscales of emotional coping, r = -0.10, and avoidance coping, r = -0.15). | Stratified by gender |
| Amirkhan  (2017) | Educational level | Coping  (CSI) | Educational level was negatively associated with inadequate coping (subscale of avoidance coping, r = -0.25). | n.a. |
| Montoya-Williams  (2020) | Educational level | Resilience  (AHRI) | Participants scored higher mean resilience scores with increasing educational level (mean scores of 11.9 (less than high school), 14.0 (less than college), 15.8 (college degree), 16.2 (more than college), range of 0-29). | n.a. |
| Batsikoura  (2021) | Educational level | Coping  (COPE) | A lower educational level was negatively associated with inadequate coping (subscale of self-blame, r = -0.22). | Age, gender, urbanization, lifestyle and anxiety |
| Holahan  (1987) | Income | Coping  (HDL) | At time point 1, family income was negatively associated with adequate coping (subscale of active-cognitive coping, r = -0.09) as well as inadequate coping (subscale of avoidance coping, r = -0.18). At time point 2, income was only negatively associated with inadequate coping (subscale of avoidance coping, r = -0.15). When additionally controlling for initial coping at time point 1, this association remained significant (r = -0.09). | Initial coping |
| Amirkhan  (2016) | Income | Coping  (CSI) | Income was negatively associated with inadequate coping (subscale of avoidance coping, r = -0.24). | n.a. |
| Tomyn  (2018) | Income | Resilience  (CD-RISC) | Participants with a household income of more than $150,000 reported higher resilience scores than participants with lower incomes of <$15,000 or $31-$60,000 (mean scores of 79.06, 72.21, and 73.90, respectively, on a scale of 0-100%). | n.a. |
| Montoya-Williams  (2020) | Income | Resilience  (AHRI) | Participants scored higher resilience scores with increasing household income (mean scores of 11.9 (<$20,000): 13.9, ($20-$49,999), 15.3 ($50-$99,999), 16.2 ($100-$149,999), and 16.5 (>$150,000), range of 0-29). | n.a. |
| Friborg  (2003) | Employment status | Resilience  (RSA) | Being employed was positively associated with resilience (subscales of personal competence, r = 0.18, social competence, r = 0.22, and family coherence, r = 0.17). | n.a. |
| Friborg  (2003) | Employment years | Resilience  (RSA) | Number of years in work was positively associated with resilience (subscales of family coherence, r = 0.17, and personal structure, r = 0.17). | n.a. |
| Anderson  (1991) | Employment arrangement | Coping  (F-copes) | Men from a dual-career employment arrangement at home (both partners working and a focus on career) used more adequate coping compared to men from a traditional employment arrangement at home (woman stays at home) (subscale of problem-focused coping/passive acceptance, mean score of 10.1 versus 7.8, range of 4-20). Women from a traditional or dual-job arrangement at home used more adequate coping than women from a dual-career arrangement did (subscale of seeking support, mean scores of 13.7, 13.7 and 10.9, respectively, range of 4-20). | Stratified by gender |
| Haan  (1964) | Socioeconomic status | Coping  (no scale) | For men, positive associations were discovered between adolescences social status and adequate coping (subscales of objectivity, r = 0.30, intellectuality, r = 0.36, and logical analysis, r = 0.32). There was no association discovered between adolescence social status and adult coping for women. Adult social status was positively associated with total adequate coping for men and women (r = 0.61 and 0.55) and the adequate coping subscales of objectivity (r = 0.46 and 0.46), intellectuality (r = 0.57 and 0.60), logical analysis (r = 0.56 and 0.58), sublimation (r = 0.38 and 0.57) and suppression (r = 0.57 and 0.32). Additionally, for men, social status during adulthood was also positively associated with the adequate coping subscales of tolerance (r = 0.44), concentration (r = 0.46), empathy (r = 0.33) and substitution (r = 0.56). | n.a. |
| Howerton  (2009) | Socioeconomic status | Coping  (MCI) | Socioeconomic status was positively associated with adequate coping (subscale of problem-focused coping, b = 0.33) and as well as with inadequate coping (subscale of emotion-focused coping, b = 0.19), while controlling for gender and ethnicity. A negative association was found between SES and inadequate coping (subscale of avoidance coping, b = -0.34). The associations remained significant while additionally controlling for experienced chronic strain (b = 0.35, 0.20, and -0.27 respectively). | Gender, ethnicity, chronic strain |
| Haan  (1964) | Social mobility | Coping  (no scale) | Both men and women made more use of adequate coping strategies when their social status was higher at time point 2 (adulthood) compared to time point 1 (adolescence) (24 years apart). For men, this was marked by using more adequate coping strategies that involved controlling impulses while for women this was marked by more adequate strategies that involved expressing their impulses. | n.a. |
| Social environment | | | | |
| De Ridder  (1995) | Marital status | Coping  (WCC) | No significant associations were discovered between marital status and coping. | n.a. |
| Matud  (2004) | Household size | Coping  (CSQ) | Number of children was positively associated with adequate coping for men (subscale of rational coping, r = 0.16) and positively associated with inadequate coping for women (subscale of avoidance coping, r = 0.15). | Stratified by gender |
| Holahan  (1987) | Family support | Coping  (HDL) | Family support was positively associated with adequate coping (subscales of active-cognitive coping, r = 0.10, and active-behavioral, r = 0.17) and negatively with inadequate coping (subscale of avoidance coping, r = -0.34) at time point 1. At time point 2, support was positively associated with adequate coping (subscale of active-behavioral coping, r = 0.09) and negatively with inadequate coping (subscale of avoidance coping, r = -0.27). When controlling for initial coping at time 1, support remained negatively associated with inadequate coping (subscale of avoidance coping, r = -0.19). | Initial coping |
| De Ridder  (1995) | Social support | Coping  (WCC) | No significant associations were discovered between perceived social support and coping. | n.a. |
| Alexander  (2001) | Social support | Coping  (WCC-R) | Perceived social support was positively associated with using adequate coping for women (subscale of seeking support, r = 0.23). | Stratified by gender |
| Pallant  (2002) | Sense of coherence | Coping  (COPE) | Respondents with a higher sense of coherence of their social world used more adequate coping (subscales of active, r = 0.37, planning, r = 0.33, reinterpretation, r = 0.22, and instrumental support coping, r = 0.10) and less inadequate coping (subscales of behavioral disengagement, r = -0.41, denial, r = -0.24, drug use, r = -0.18, and mental disengagement, r = -0.11). Additionally, for women sense of coherence was also positively associated with other adequate strategies (emotional support, r = 0.20, and humor, r = 0.16). Differences were found between men and women and the effect of sense of coherence on coping. The positive associations with adequate coping were stronger for women (subscales of active coping, r = 0.43 versus 0.26, and planning, r = 0.38 versus 0.23) as well as the negative associations with inadequate coping (subscales of behavioral disengagement, r= -0.43 versus -0.35, and denial, r = -0.25 versus -0.21) except for one subscale (drug use, r = -0.15 versus -0.23). | Socially desired responding |
| Roussi  (2006) | Sense of community | Coping  (SACS) | A higher perceived sense of community was positively associated with adequate coping (subscales of social joining, r = 0.21) and negatively associated with inadequate coping (subscales of aggressive action, r = -0.18, and antisocial action, r = -0.27). | n.a. |
| Capanna  (2013) | Social connectedness | Resilience  (RSA) | Social connectedness was positively associated with the total resilience score (r = 0.48) as well as the resilience subscales: perception of self, r = 0.51, planned future, r = 0.50, social competence, r = 0.58, family cohesion, r = 0.34, and social resources, r = 0.42. | n.a. |
| Yu  (2021) | Acculturation | Resilience  (CD-RISC) | A higher immersion of the dominant culture is positively associated with resilience (r = 0.32). No significant effect was found for immersion of ethnic cultures. | n.a. |
| Psychosocial well-being and life experiences | | | | |
| Matud  (2004) | Chronic strain/stress | Coping  (CSQ) | Experiencing chronic stress was positively associated with inadequate coping for women (subscale of emotional coping, r = 0.22). | Stratified by gender |
| Howerton  (2009) | Chronic strain/stress | Coping  (MCI) | Experiencing chronic strain was positively associated with adequate coping (subscale of problem-focused coping, b = 0.01) and inadequate coping (subscale of avoidant coping, b = 0.04), while controlling for gender, ethnicity and SES. | Gender, ethnicity, SES |
| Alexander  (2001) | Daily strain/stress | Coping  (WCC-R) | Experiencing (parenting) strain was positively associated with using adequate coping for both men and women (subscales of problem-focused coping, r = 0.50 and 0.22, and seeking support, r = 0.46 and r = 0.21) as well as using inadequate coping (subscale of emotional-focused coping, r = 0.48, and 0.60). The associations with adequate coping were stronger for men, while the association with inadequate coping was stronger for women. | Stratified by gender |
| Matud  (2004) | Daily stress/strain | Coping  (CSQ) | Experiencing minor daily stress was positively associated with inadequate coping for women (subscale of emotional coping, r = 0.10). | Stratified by gender |
| Vingerhoets  (1990) | Characteristics of stressor | Coping  (WCC-R) | The severity of daily stressors was positively associated with adequate coping for men and women (subscale of positive thinking/growth/humor, β = 0.13 and 0.12) and negatively related with inadequate coping (subscale of distancing, β = -0.18 and -0.17). For men, severity of daily stressors was also positively associated with another subscale of adequate coping (planning and rational action, β = 0.14). For women, severity of daily stressors was also positively associated with another subscale of inadequate coping (day dreaming and fantasizing, β = 0.12). The unpleasantness of daily stressors was negatively associated with inadequate coping for men and women (subscale of distancing, β = -0.13 and -0.13), as well as positively associated with adequate coping for women (subscale of positive thinking/growth/humor, β = 0.14). | Depression and anxiety symptoms Stratified by gender |
| De Ridder  (1995) | Characteristics of stressor | Coping  (WCC) | The appraised stressfulness of a situation to cope with was positively associated with adequate coping (subscales of positive reappraisal, r = 0.21, seeking social support, r = 0.27, planful problem solving, r = 0.37, and accepting responsibilities, r = 0.36). The appraised manageability of a situation to cope with was also positively associated with adequate coping (subscales of positive reappraisal, r = 0.19), seeking social support, r = 0.24, and accepting responsibility, r = 0.34). | n.a. |
| Irion  (1987) | Domain of stressor | Coping  (WCC) | When faced with threatening situations compared to challenging situations, young adults used more inadequate as well as adequate coping strategies (inadequate: subscales of escape-avoidance, mean score of 0.97 versus 0.88, hostile reaction, mean score of 0.71 versus 0.65, distancing, mean score of 1.09 versus 0.95, self-blame, mean score of 1.65 versus 1.39, and altruism, mean score of 1.40 versus 1.39. Adequate: subscales of confrontive coping, mean score of 1.11 versus 0.83, planful-problem solving, mean score of 1.76 versus 1.73, seeking social support, mean score of 1.54 versus 1.44, and self-controlling, mean score of 1.22 versus 1.21, range of 0-3), with the exception of one adequate subscale of coping (positive reappraisal, mean score of 1.38 versus 1.52, range of 0-3). | n.a. |
| Harnash  (2000) | Domain of stressor | Coping  (no scale) | Using inadequate coping was higher when being faced with interpersonal stressors compared to transition related stressors (subscale of avoidance coping, mean score of 11.80 versus 11.08, range of 4-16). Using adequate coping was higher when faced with transition related stressors compared with interpersonal and illness related stressors (subscale of reappraisal coping, mean scores of 12.48 versus 10.92 and 10.85, range of 4-16) and when facing role strain stressors compared to interpersonal and illness related stressors (subscale of reappraisal coping, mean scores of 12.00 versus 10.92 and 10.85, range 4-16). Using adequate coping was lower when facing interpersonal stressors when compared to transition and role strain related stressors (subscale of active behavioral coping, mean scores of 11.27 versus 11.99 and 11.89, range of 4-16). Using adequate coping was higher when facing transition related stressors compared to interpersonal, role strain and illness related stressors (subscale of seeking support coping, mean scores of 13.09 versus 11.91, 11.89 and 11.54, range of 4-16). Using adequate coping was highest when facing illness related stressors compared to interpersonal, transition and role strain stressors (subscale of religion coping, mean scores of 10.28 versus 7.49, 7.91, and 7.28, range of 4-16). | n.a. |
| Simeon  (2007) | Childhood trauma | Resilience  (DSQ) | The overall, composite measure of childhood trauma was negatively associated with resilience (r = -0.43). Two subscales of childhood trauma were negatively associated with resilience; emotional neglect (r = -0.39) and physical abuse (r = -0.37). | Age, gender |
| Amirkhan  (2017) | Childhood trauma | Coping  (CSI) | Childhood trauma was positively associated with inadequate coping (subscale of avoidance coping, r = 0.30). The significant effect remained while controlling for confounding factors. | Education, income |
| Amirkhan  (2017) | Lifetime trauma | Coping  (CSI) | Lifetime trauma was positively associated with inadequate coping (subscale of avoidance coping, r = 0.30). In a hierarchical regression with the variables education, income and childhood trauma, lifetime trauma added no further explanatory power in predicting the use of the avoidance coping. | Education, income, childhood trauma |
| Holahan  (1987) | Negative life events | Coping  (HDL) | Experiencing negative life events was positively associated with adequate coping (subscales of active-cognitive, r = 0.12, and active-behavioral, r = 0.11) and inadequate coping (subscale of avoidance coping, r = 0.18) at time point 1. At time point 2, the effects remained (respectively r = 0.21, 0.20, and 0.25). When controlling for initial coping at time point 1, the effects remained significant at time point 2 (respectively r = 0.20, r= 0.17 and r = 0.24). | Initial coping |
| Matud  (2004) | Life events (number, uncontrollability, undesirability) | Coping  (CSQ) | The number of life events was positively associated with inadequate coping for women (subscale of emotional coping, r = 0.11). The uncontrollability of life events was negatively associated with adequate coping for women (subscale of rational coping, r = -0.11) and positively associated with inadequate coping for both women and men (subscale of emotional coping, r = 0.21 and 0.20). The undesirability of life events was negatively associated with adequate coping for women (subscale of rational coping, r = -0.10) and positively associated with inadequate coping for women and men (subscale of emotional coping, r = 0.22 and 0.17). | Stratified by gender |
| Vingerhoets  (1990) | Depression and anxiety symptoms | Coping  (WCC-R) | Higher scores on the symptoms checklist for psychosocial disturbances were positively associated with inadequate coping for both men and women (subscales of self-blame, β = 0.33 and 0.33, daydreaming and fantasizing, β = 0.22 and 0.18, and wishful thinking/emotionality, β = 0.23 and 0.14) and negatively associated with adequate coping (subscale of positive thinking/growth/humor, β = -0.21 and -0.16). For men, it was also negatively associated with adequate coping (subscale of planning and rational actions, β = -0.18). | Stratified by gender |
| Matud  (2004) | Depression and anxiety symptoms | Coping  (CSQ) | Experiencing psychological distress was negatively associated with adequate coping for women and men (subscale of rational coping, r = -0.14 and -0.11) and positively associated with inadequate coping (subscale of emotional coping, r = 0.38 and 0.32). | Stratified by gender |
| Batsikoura  (2021) | Anxiety symptoms | Coping  (COPE) | Experiencing negative feelings was positively associated with adequate coping (subscale of emotional/instrumental support, r = 0.12), as well as with inadequate coping (subscale of denial/disengagement, r = 0.15), but also negatively with adequate coping (subscale of reframing/humor, r = -0.10). Experiencing positive feelings was positively associated with adequate coping (subscales of emotional/instrumental support, r = 0.08, reframing/humor, r = 0.11, and acceptance/planning, r = 0.08). It was negatively associated with inadequate coping (subscale of denials/disengagement, r = -0.06). Having strong emotional feelings was positively associated with adequate coping (subscales of reframing/humor, r = 0.11, and acceptance/planning, r = 0.08) and inadequate coping (subscale of self-blame, r = 0.14). | Age, gender, educational level, urbanization, lifestyle, |
| De Ridder  (1995) | Satisfaction with life | Coping  (WCC) | A feeling of satisfaction with one’s life was negatively associated with adequate coping (subscale of accepting responsibilities, r = -0.20). | n.a |
| Matud  (2004) | Satisfaction with work(role) | Coping  (CSQ) | Dissatisfaction with one’s role (at work or at home in case of unemployment) was negatively associated with adequate coping for both women and men (subscale of rational coping, r = -0.08 and -0.11) and positively associated with inadequate coping (subscale of emotional coping, r = 0.24 and 0.20). | n.a. |
| Location | | | | |
| Roussi  (2006) | Urbanization | Coping  (SACS) | A cluster analysis revealed two clusters of coping strategies in urban areas. Firstly, a cluster of assertive and indirect coping combined with antisocial and aggressive coping. Secondly a cluster consisting of cautious coping, social joining and seeking support. For rural areas, also two clusters were discovered. Firstly, a cluster of assertive and indirect coping combined with cautious coping, social joining and seeking support. Secondly, a cluster of antisocial and aggressive coping. When comparing the two areas, it is seen that in urban areas assertive and indirect coping is combined with antisocial coping strategies whereas in rural areas assertive and indirect coping is combined with prosocial coping strategies. | n.a. |
| Batsikoura  (2021) | Urbanization | Coping  (COPE) | Living in a smaller town was associated with using adequate coping (subscales of emotional/instrumental support, r = 0.43, and positive reframing/ humor, r = 0.33). | Age, gender, educational level, lifestyle and anxiety |
